# Disinfection and Disinfectants

**Published:** 1902-01

**Authors:** 


Disinfection and Disinfectants. A Treatise Upon the Best Known Disinfectants; their Use in the Disinfection of Disease Germs, with Special Instruction for their Application in the Commonly Recognized Infectious and Contagious Diseases. By H. M. Bracken, M. D., Professor of Materia Medica and Therapeutics, University of Minnesota; Secretary and Executive Officer of the Minnesota State Board of Health. Pages, 90. Price, $1.00. Published by The Trade Periodical Company, Chicago. 1900.

This is a handy and thoroughly practical little volume. Every man's peculiar views are not given, but demonstrated methods such as are in vogue among the most celebrated authorities are embodied in the work. It is a useful little book to have about one's office.

